# Supplementary material for: A Comprehensive Assessment of Ultraviolet-Radiation-Induced Mutations in Flammulina filiformis Using Whole-Genome Resequencing
Source: J Fungi (Basel). 2024 Mar 20;10(3):228. doi: 10.3390/jof10030228 (PMC10971301; doi:10.3390/jof10030228)
Supplement: Supplementary file 1 [file jof-10-00228-s001.zip › Supplementary Material S8/KEGG annotation/out/64381550635650.os/KO/out_map/map04510.html]

KEGG PATHWAY: Focal adhesion - Reference pathway


|  |  |
| --- | --- |
| **Focal adhesion - Reference pathway** |  |

[
Pathway menu
| Organism menu
| Pathway entry
| Show description
| User data mapping
]

Cell-matrix adhesions play essential roles in important biological processes including cell motility, cell proliferation, cell differentiation, regulation of gene expression and cell survival. At the cell-extracellular matrix contact points, specialized structures are formed and termed focal adhesions, where bundles of actin filaments are anchored to transmembrane receptors of the integrin family through a multi-molecular complex of junctional plaque proteins. Some of the constituents of focal adhesions participate in the structural link between membrane receptors and the actin cytoskeleton, while others are signalling molecules, including different protein kinases and phosphatases, their substrates, and various adapter proteins. Integrin signaling is dependent upon the non-receptor tyrosine kinase activities of the FAK and src proteins as well as the adaptor protein functions of FAK, src and Shc to initiate downstream signaling events. These signalling events culminate in reorganization of the actin cytoskeleton; a prerequisite for changes in cell shape and motility, and gene expression. Similar morphological alterations and modulation of gene expression are initiated by the binding of growth factors to their respective receptors, emphasizing the considerable crosstalk between adhesion- and growth factor-mediated signalling.

|  |  |
| --- | --- |
| Reference pathway | 100% |
